# Design of a Reconfigurable Wall Disinfection Robot

**DOI:** 10.3390/s21186096

**Published:** 2021-09-11

**Authors:** Ash Wan Yaw Sang, Chee Gen Moo, S. M. Bhagya P. Samarakoon, M. A. Viraj J. Muthugala, Mohan Rajesh Elara

**Affiliations:** Engineering Product Development Pillar, Singapore University of Technology and Design, 8 Somapah Rd, Singapore 487372, Singapore; ash_wan@mymail.sutd.edu.sg (A.W.Y.S.); cheegen_moo@sutd.edu.sg (C.G.M.); bhagya_samarakoon@mymail.sutd.edu.sg (S.M.B.P.S.); rajeshelara@sutd.edu.sg (M.R.E.)

**Keywords:** cleaning robotics, reconfigurable robotics, wall cleaning, wall following

## Abstract

During a viral outbreak, such as COVID-19, autonomously operated robots are in high demand. Robots effectively improve the environmental concerns of contaminated surfaces in public spaces, such as airports, public transport areas and hospitals, that are considered high-risk areas. Indoor spaces walls made up most of the indoor areas in these public spaces and can be easily contaminated. Wall cleaning and disinfection processes are therefore critical for managing and mitigating the spread of viruses. Consequently, wall cleaning robots are preferred to address the demands. A wall cleaning robot needs to maintain a close and consistent distance away from a given wall during cleaning and disinfection processes. In this paper, a reconfigurable wall cleaning robot with autonomous wall following ability is proposed. The robot platform, Wasp, possess inter-reconfigurability, which enables it to be physically reconfigured into a wall-cleaning robot. The wall following ability has been implemented using a Fuzzy Logic System (FLS). The design of the robot and the FLS are presented in the paper. The platform and the FLS are tested and validated in several test cases. The experimental outcomes validate the real-world applicability of the proposed wall following method for a wall cleaning robot.

## 1. Introduction

### 1.1. Background

Ongoing zoonotic pathogens have emerged since 2019 and continue to challenge societal efforts to fight against the highly infectious spread of SARS-CoV-2. This disease can be transmitted either from human-to-human, from contaminated objects/surfaces-to-human, or airborne infections [1,2,3,4]. Almost all countries are facing difficulties due to this pandemic. While countries are making their best efforts to reopen their borders, the recuperation of human mobility will potentially lead to the outbreak of new waves with new variants [5]. During the incubation period of SARS-CoV-2, the virus carrier may not reflect any symptoms that extend the virus’ spread from the carrier’s daily environmental and social interactions. Contaminated surfaces can be objects or especially public or common areas. These contaminated areas bear the highest risk of infecting new victims and hence affecting socioeconomic stability [6,7].

The current pandemic is not our first viral disease [8] and will also not be the last. Indirect transmissions can be due to the contamination of public surface areas from an infected person [9]. In order to reduce indirect transmissions, disinfection processes in public areas can be conducted by spraying disinfectants, dispensing sanitisers, ultraviolet light or physical cleaning. The floor and wall areas contribute to a large part of a typical indoor environment and can be an indirect mode of transmission for the spread of the COVID-19 virus or any other diseases [10,11]. Despite having restrictive measures in place, disinfecting strategies should also be in place to better prepare for the new normal [12,13].

### 1.2. Existing Technologies

#### 1.2.1. Disinfection Methods and Mechanism

Disinfection operations are creating risk for frontline workers [14] as these operations are manual. Manual labourers play an essential role in this viral crisis as they are also required in other parts of the pandemic management system. Hence, the challenges of manual disinfection methodologies have to be readdressed and reconsidered. Automating this process with robots can reduce disease transmission between healthcare workers and also provide better hygiene in their environments [15,16,17,18,19,20]. To effectively disinfect potentially contaminated areas, robots can serve to contribute towards an efficacious containment of the infectious disease.

Disinfection methods are found in the literature together with the development of semi and fully-teleoperated robots [21,22]; a surface disinfection robot can therefore be adopted. The development of teleoperated robots may provide a telepresence for frontline workers as part of a solution to reduce the risk of infection. This can be seen to be inadequate in contrast to autonomous systems to ease the commitment from human operators. In a combined system of a master–slave with human-in-the-loop, the system may be unstable. Therefore, an autonomous disinfection robot is preferable. Comparatively, walls can be easily contaminated with viruses in high-risk areas such as hospitals. The disinfection process should require robots to operate along the walls of high-risk indoor places autonomously.

Most of the literature discusses automation methods by using robots equipped with sprayers or, mostly, UV lamps [16,18,21]. The exploitation of UV technologies as a method for surface disinfection was highly efficient (up to 99.99 percent). One of the three main classifications of UV and UV-C radiation (range 100 to 280 nm), is considered the most effective disinfection class among other UV class ranges in viral inactivation as discussed by Houser [23]. It is harmful to viruses and bacteria. In the case of COVID-19, UV-C damages its RNA sequence, making it unable to replicate. In application, it is proportional to the duration of exposure and intensity. Hence, robots can either be designed with higher UV-C intensity lamps or with controlled motion time functions. UV-C equipped robots are not preferable in a human-shared environment as UV-C radiation is harmful to human skin and eyes. Traditional surface decontamination methods include mainly fogging, fumigation, electrostatic spraying and UV light [24]. More commonly, disinfectant sprayers and sanitiser dispensers are utilised in COVID-19 management [8,15].

Wall cleaning mechanisms are typically wall-climbing robot cleaners as compared to their manipulator cleaning counterparts [25,26,27,28]. Wall robots’ mobility on the walls is mostly performed via suction (either with wheel or legs), thrust or auxiliary mechanisms (i.e., rope, scaffold etc.). These wall cleaners moving on vertical surfaces are constrained by mostly consistent features; overcoming obstacles and intricate wall features is challenging. Two-DOF manipulators were proposed; the distance and angle of the end-effectors can be controlled by a ball-screws mechanism, proposed by Kim et al. [29], and by an eight joint manipulator proposed by Joo et al. [30]. As end-effectors, the robot’s cleaning module is typically equipped with various types and combinations of brushes, squeegees and scrapers. Most suction robots are equipped with tools for dry cleaning or diatomite methods, while wire/roped robots are equipped with water cleaning tools [25]. Examples of common cleaning methods of cleaning robots include the TITO 500 [31] and the CAFE robot [32], which use brushes and water sprays. Most of these cited works discuss the development of wall cleaning robots designed for high rise buildings’ outer walls. There are fewer discussions regarding the development of indoor wall cleaning robots.

#### 1.2.2. Wall Following Methods

Autonomous wall-following ability is essential for a wall cleaning mobile robot to maintain a proper clearance with the wall. Furthermore, a wall cleaning robot should have the ability to work in an unknown environment. In this regard, online-decision making ability is preferred over offline planners [16,33]. Therefore, the wall-following method for a wall cleaning robot should have online decision-making ability.

Many popularly used general autonomy algorithms are generalized models for autonomy implementation. Some of the common algorithms are line-tracing algorithms [34,35], SLAM [36,37], probabilistic [38,39] and Artificial Potential Fields [40]. These algorithms may not be suitable, as trace-tracing algorithms require the set-up and maintenance of visibly traceable lines, and other methods optimize the shortest path/energy approach. The shortest path approach is not suited for wall-following methods as the robot should aim to cover as much area as possible, and it should always be navigating down the walls of the facilities closely, which is not provided by the waypoints of common approaches.

The use of Proportional–Integral–Derivative (PID) controllers for wall-following has been studied in [41,42]. However, the targeted wall was straight, and these proposed PID controllers were ineffective in coping with curved or slanted walls. Furthermore, the controller gains need to be tuned for different set points, which hinders the usability of a reconfigurable wall cleaning mechanism. The authors of [43,44] investigated the performance of different neural networks for wall-following. The robot’s classification ability corresponding to a set of sensor readings is used for the performance analysis. However, the investigations are limited to validation through a dataset; no real-world validation or simulation has been conducted. Therefore, the validity of the proposed method is doubtful in real-world applications. In addition to that, the requirement of a comprehensive dataset that covers most probable scenarios is increasingly complicated and dependent on the sensor arrangement of a robot.

Fuzzy logic systems tuned through optimization have been widely used due to their ability to cope with uncertainties. In this regard, various methods for tuning fuzzy logic systems have been investigated, including ant-colony optimization [45], differential evolution [46], bee-colony optimization [47,48], and reinforcement learning. Furthermore, the use of interval type 2 fuzzy logic systems tuned by these techniques has also been studied [49,50]. These methods are capable of effectively taking control actions to follow walls after training on environments. However, the main shortcoming of these methods is the requirement for retraining when changing the reference distance from the wall. Thus, the methods are ineffective for a wall cleaning robot with different end-effectors where the distance with the wall has to be remodelled for different tools. Furthermore, generalization of the training is also a concern since it requires training in many environments.

Several fuzzy logic systems based on expert knowledge have been developed for the wall-following features of mobile robots. The work [51] proved that a fuzzy logic system could have better performance than a PID controller in terms of wall-following. Another similar sort of fuzzy logic controller defined based on expert knowledge for wall-following has been presented in [52]. A system consisting of three individual fuzzy logic systems switched per the scenario of wall-following has been proposed in [53,54]. The system analyzes the range sensor information and determines the current scenario as a straight, left corner, and right corner wall. The fuzzy logic system designed for each of these scenarios is then selected for generating the control action. The use of condition-based fuzzy logic system selection is susceptible to sensor noises. All the fuzzy logic systems cited above are capable of establishing the wall-following ability of a robot without requiring training. However, all these systems are tailored for a specific clearance distance with a wall, and the membership functions have to be redesigned to allow the systems to work with different reference distances. Thus, the cited systems are not convenient for a wall cleaning robot with a reconfigurable end-effector. The work [55] proposed a fuzzy logic system that can adapt to different reference distances for a wall disinfection robot that eliminates the limitations of all the other wall following methods discussed above. Hence, it is the work that is most relevant for this paper. However, the scope of the work is limited to simulations, and experiments with a real robot deployment have not been conducted. The limitations of the existing wall-following methods discussed above are summarized in Table 1.

#### 1.2.3. Reconfigurable Robotics

The trend of reconfigurable robots is increasing as they are set to provide high versatility of functions by adopting various shapes and forms. The types and range of applications can vary to fit the demands of users. This makes them highly attractive to facilities managers of, for example, hospitals and airports, due to the high cost–benefit ratio. Reconfiguration [56] can be classified in three ways; intra-, inter-, or nested-reconfiguration. Intra-reconfigurable robots possess the ability to transform internally without involving any additional module, while inter-reconfiguration may consist of the assembly or reassembly of homogeneous or heterogeneous robot modules as a single unified platform. Nested-reconfigurable robots involve both intra- and inter-reconfigurable functions integrated into a single robot platform.

Adopting inter-reconfigurable robots is advantageous for hospitals where robot platforms can converge into a single platform with inter-reconfigured modules for different applications. Autonomous reconfigurable partners can help to ease hospital operations for staff and include more value-adding tasks into the scope of their jobs. Reconfigurable robots have yet to fulfil the promise of versatility [57,58]. It has been implied in studies that reconfiguration has to be useful for end-users for it to be considered versatile.

### 1.3. Aims and Contributions

This paper presents and proposes the implementation of an autonomous wall-following ability in a reconfigurable wall-cleaning robot. The aim of the proposed solution is to, firstly, unveil a new autonomous inter-reconfigured robot application to a hospital robot’s range of versatility and, secondly, a proof of real-world integration that has not previously been established. Prior to this development, the algorithm had not been tested or verified in any physical robot platform. Real-world application refers to the physical validation of the usability of the methodological approach and the appropriate use of the approach. In this paper, the practical use of the approach is in the context of disinfection operations. The physical robot is to be observably moving along a targeted wall while maintaining a fixed distance. The wall-following algorithm is a Fuzzy Logic System (FLS) that dictates the robot’s reference linear and angular velocities during operations. The proposed exploitation of this method is to enable robots for wall-cleaning or disinfection tasks to be used in public areas at high risk of contaminants. The experiments have considered a range of typical wall features and evaluate the capabilities of the robot’s wall-following behaviours.

The following sections of the paper include a technical introduction of the utilized robot platform and wall-following methodology in Section 2 and Section 3. Subsequently, in Section 4, the paper details the experimental set-up then presents the experimental results and discussions. The paper concludes the research findings in adopting the autonomous function in robot mobility for wall-following in Section 5.

## 2. Robot Platform

### 2.1. Overview

In this paper, the Wasp platform is equipped to demonstrate the wall-following behaviour, specifically in the example of wall cleaning by integrating a wall cleaning module into the platform. This section briefly describes the motivation behind the Wasp platform, followed by the mechanical design, electrical system, and kinematics modelling of the Wasp platform.

Firstly, Wasp is a robot that is expected to be developed into a highly versatile robot platform in healthcare settings in collaboration with Changi General Hospital (CGH) in Singapore. With the ability to reconfigure, the application of Wasp can be, but is not limited to, patient handling, logistics, cleaning and surveillance. One exemplary application of the Wasp platform is to be utilized as a logistic robot to transport different types of trolleys after reconfiguration. Presently, numerous robots from different developers have been utilized in CGH, each with its rated payload and dimensions, mainly to automate the trolley transportation process. However, due to product diversification, each of these robots has its unique specification, communication protocols and charging platforms, which exponentially reduce the ease of centralized management of these robots. What is more, these robots are designed as a single module to operate independently with their distinct mechanism. In other words, each of these robots has limited capability to handle a particular type of trolley, and different robots are needed for different types of trolleys. As a result, the demand for robots with different specifications and functions is overwhelming, and this opposes the economic benefits as more efforts and resources are needed to manage these robots. Under such circumstances, nested-reconfigurable robots are recommended.

Research on reconfigurable robots was initiated in the 1980s. It originated from modular robotics, which refers to a family of robotics systems made of interconnected individual robots. These individual robots, also referred to as modules, are relatively small in size and are able to perform tasks independently. Currently, this area is progressing in autonomy development. When needed, these robots can be interconnected (module-to-module) through docking interfaces to form a larger physical structure and gain the advantage of size to cope with physically larger tasks. The ability of these modular robots to morph into different structures has earned themselves the name of reconfigurable or self-reconfigurable robots, depending on the autonomy level associated with the process [59,60]. Traditionally, reconfigurable robots used to be classified into lattice, chain and hybrid types until a group of researchers proposed a new classification concept, the intra-, inter- and nested-reconfigurable robots in [56]. Intra-reconfigurable robots possess the ability to transform internally without involving an external module, while inter-reconfigurable robots have the ability to assemble external modules to form a new robot. Nested-reconfigurable robots involve both intra- and inter-reconfigurability being integrated into the same robot. With the promise of a high degree of versatility, robustness and modularity, nested-reconfigurable robots would be an ideal solution to the dynamic demands of a typical healthcare environment. Thus, the Wasp platform is designed as a nested-reconfigurable robot; not only is it expected to transport different types of trolleys after intra-reconfiguration, but also to perform other specific tasks such as wall cleaning after inter-reconfiguration with an external wall cleaning module.

### 2.2. Mechanical Design

**Figure 1:**
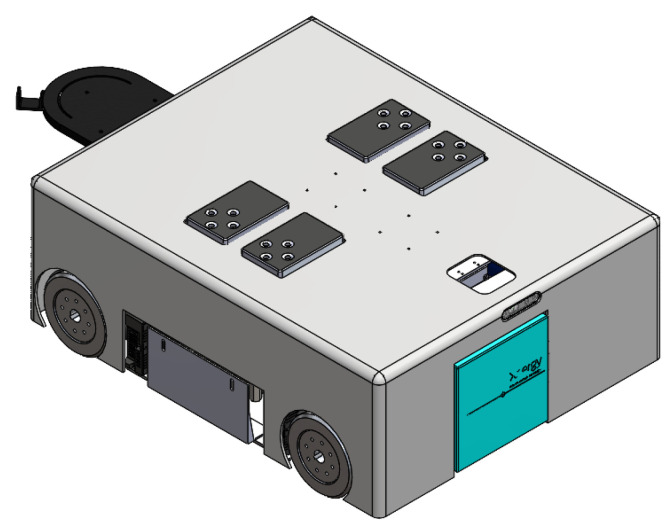
External view of Wasp platform.

**Figure 2:**
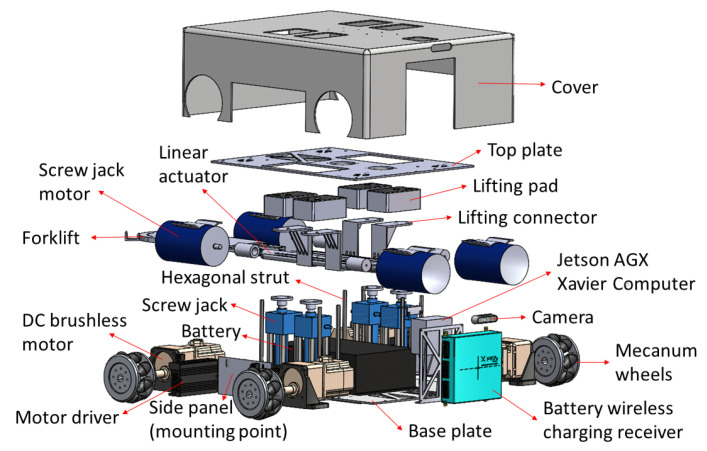
Exploded view of Wasp platform.

As shown in Figure 1, the Wasp platform has a rectangular geometry. Internally, its mainframe is comprised of a top plate and a base plate, both made of stainless steel. Ten hexagonal struts are used to connect the top and base plates to create a clearance height of 266 mm to accommodate both mechanical and electrical parts of the platform. Referring to Figure 2, there are four units of screw jack with motors and a unit of forklift attached to a linear actuator for the lifting mechanism and towing mechanism, respectively. These two mechanisms enable the platform to be intra-reconfigurable to handle different trolley types in CGH. In lifting mode, the Wasp platform lifts the trolleys with sufficient ground clearance from underneath using screw jacks. Meanwhile, in towing mode, the Wasp platform tows the trolleys with smaller ground clearance using the forklift extended out of the robot body as shown in Figure 1. Besides, four Mecanum wheels are used for robot locomotion due to their high manoeuvrability. By driving these wheels with different combinations of speed and direction using DC brushless motors, the Wasp platform is able to move freely in any direction at any instance without changing its orientation. Such omnidirectional mobility allows the platform to perform tasks effectively in constrained environments (i.e., obstacles and narrow spaces) [61].

**Figure 3:**
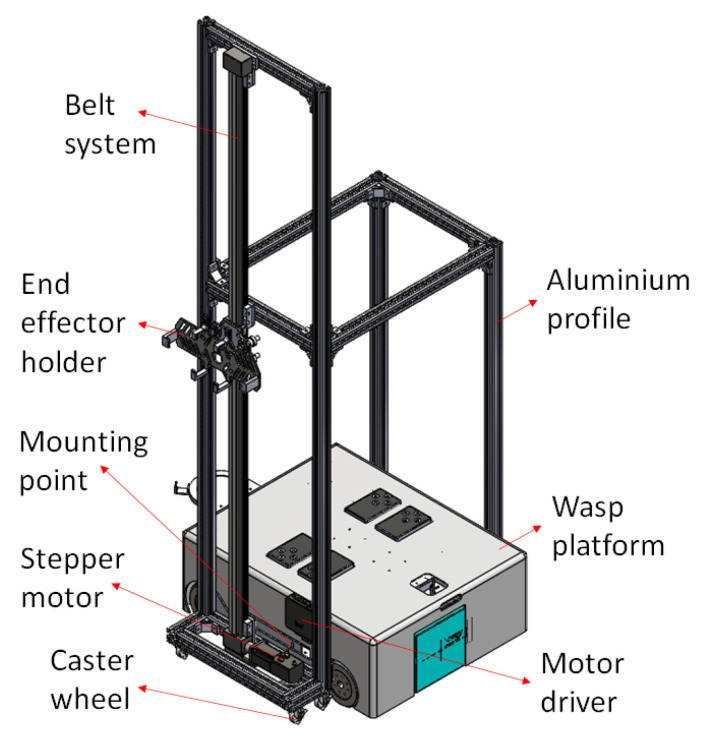
Integration of wall cleaning module with Wasp platform.

The exploded view in Figure 2 shows a side panel available in the Wasp platform as a mounting point for an external module. In this paper, a wall cleaning module is particularly described to assist in demonstrating the wall-following behaviour of the Wasp platform. As shown in Figure 3, the wall cleaning module framework is constructed using aluminium profiles while caster wheels are used for its mobility. Furthermore, a belt system is adopted vertically for the up-down movement of the end-effector, with the end-effector holder installed on the belt system table. Figure 3 also shows an illustration of the wall cleaning module mounted on the platform. Additional and other on-board functions and design architecture are not further elaborated, given the scope of this paper. The assembly of this external wall cleaning module onto the Wasp platform demonstrates the inter-reconfigurability of the Wasp platform as mentioned earlier.

### 2.3. Power System and Electronics

**Figure 4:**
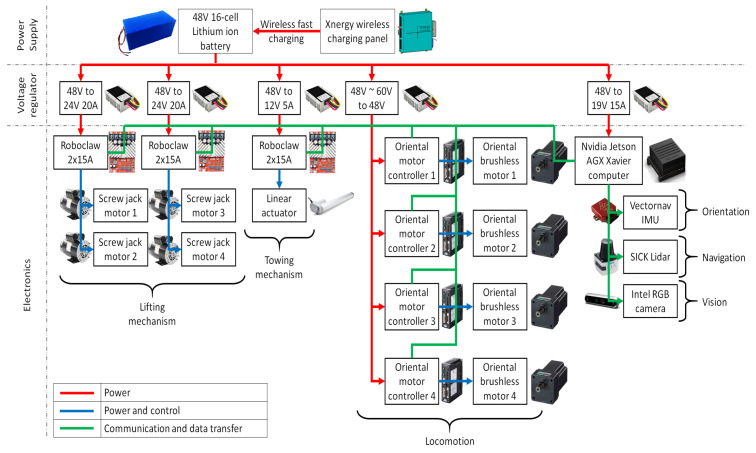
Electrical system diagram of Wasp platform.

As shown in Figure 4, the Wasp platform carries a rechargeable 16-cells Lithium-ion battery as its main power supply. This battery supplies 48VDC to the platform and is charged wirelessly using the Xnergy wireless charging panel. Wireless charging mode with fast charging technology from Xnergy enable the Wasp platform to be fully charged in 50 min, and at the same time, it is free from the need for precise docking required by wired connecting chargers. Next, to power up the electronics with different power ratings, voltage regulators are used to step down 48 V to several voltage levels such as 24 V, 19 V and 12 V. Within these electronics, Roboclaw controllers (Roboclaw 2 × 15A Dual Channel Motor Controller, https://www.basicmicro.com/RoboClaw-2x15A-Motor-Controller_p_10.html accessed on 3 September 2021) are used to power and control the screw jack motors (Jacton 24 V DC motor with JTC series screw jack, part number JTC5-UK-70-H-I-C-PP, http://www.jacton-screwjacks.com/web/2018/JTCSeriesCubicScrewJacks_1114/238.html accessed on 3 September 2021) and the linear actuator (12” Mini Linear Actuator, https://www.firgelliauto.com/products/mini-linear-actuator accessed on 3 September 2021) for the lifting and towing mechanisms, respectively, while Oriental motor controllers are used to power and control the Oriental brushless motors (BLV640NM50S, 400W(1/2 HP) Brushless DC Motor Speed Control System, https://catalog.orientalmotor.com/item/blv-series-brushless-dc-motor-speed-control/400w-blv-series-brushless-dc-motors/blv640nm50s-3 accessed on 3 September 2021) to drive Mecanum wheels for Wasp platform locomotion. On the other hand, an Nvidia Jetson AGX Xavier computer is used as the central processing unit for its capability for graphics processing. Ubuntu 16.04 LTS is installed as the operating system while Robot Operating System (ROS) is used as the framework for writing the robot software. Specifically, a custom made Microsoft C++ code is implemented to run the proposed FLS in this study. LiDAR readings are collected and segmented into two main inputs for FLS, which then outputs command velocity to control the motion of the robot platform. Lastly, the Vectornav IMU sensor (VectorNav VN-100, https://www.vectornav.com/products/detail/vn-100 accessed on 3 September 2021), SICK LiDAR (SICK 2D LiDAR sensor TiM581-2050101, https://www.sick.com/be/en/detection-and-ranging-solutions/2d-lidar-sensors/tim5xx/tim581-2050101/p/p619344 accessed on 3 September 2021) and Intel RGB camera (Intel^®^ RealSense^™^ Depth Camera D435i, https://store.intelrealsense.com/buy-intel-realsense-depth-camera-d435i.html accessed on 3 September 2021) are connected to the computer for Wasp platform orientation, navigation and vision purposes, respectively.

### 2.4. Kinematics Modeling

**Figure 5:**
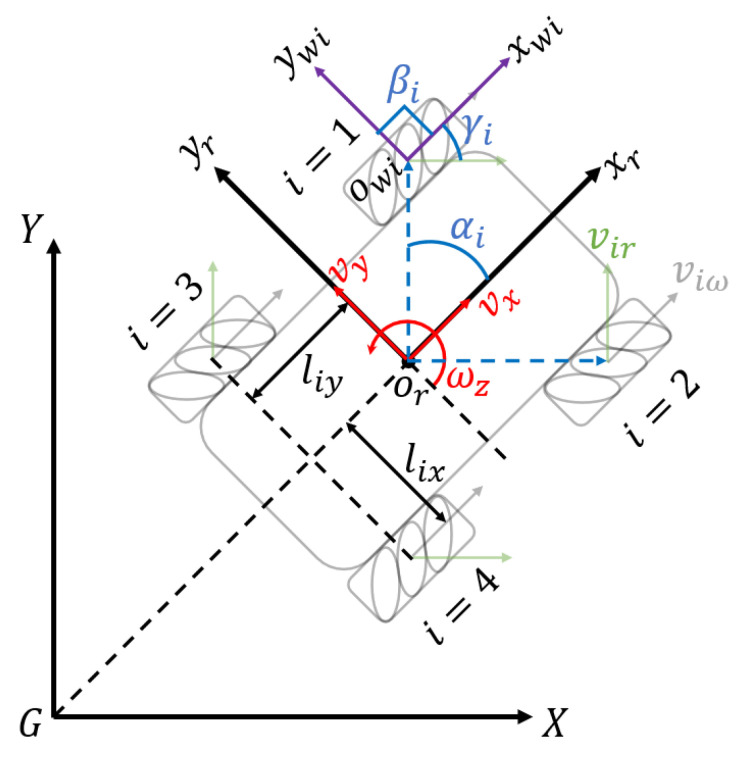
Kinematics model of Wasp platform.

As a typical four-wheel Mecanum drive robot, the kinematics model of the Wasp platform is shown in Figure 5. The symbols are defined as below:

- X, G, Y: The inertial frame.
- $x_{r},o_{r},y_{r}$: The Wasp platform base frame. $o_{r}$ is the center of robot base.
- $x_{wi},o_{wi},y_{wi}$: The coordinate system of *i*th wheel. $o_{wi}$ is the wheel center point.
- $\alpha_{i}$: The angle between $o_{r}o_{wi}$ and $x_{r}$.
- $\beta_{i}$: The angel between $x_{wi}$ and $y_{wi}$.
- $\gamma_{i}$: The angle between $v_{ir}$ and $v_{iw}$.
- $v_{ir}$: The velocity of passive rollers in *i*th wheel.
- $v_{i\omega }$: The velocity of *i*th wheel correspond to wheel revolution.
- $l_{ix}$: Half the distance between front wheels or rear wheels.
- $l_{iy}$: Half the distance between front wheel and rear wheel.
- $v_{x}$: Longitudinal velocity of Wasp platform.
- $y_{y}$: Transversal velocity of Wasp platform.
- $\omega_{z}$: Angular velocity of Wasp platform.

Assuming that wheel slippage on the ground is negligible, and all the wheels are the same size with radius *r*, the inverse kinematics equation of the Wasp platform is shown in Equation (1), while the forward kinematics equation is shown in Equation (2), with $T^{+}$ as the transpose matrix of the Jacobian matrix of the inverse kinematics equation.

(1) $$\left[\begin{array}{c} \omega_{1}\\ \omega_{2}\\ \omega_{3}\\ \omega_{4} \end{array}\right]=\frac{-1}{r}\left[\begin{array}{ccc} \frac{\cos(\beta_{1}-\gamma_{1})}{\sin\gamma_{1}} & \frac{\sin(\beta_{1}-\gamma_{1})}{\sin\gamma_{1}} & \frac{l_{1}\sin\left(\beta_{1}-\gamma_{1}-\alpha_{1}\right)}{\sin\gamma_{1}}\\ \frac{\cos(\beta_{2}-\gamma_{2})}{\sin\gamma_{2}} & \frac{\sin(\beta_{2}-\gamma_{2})}{\sin\gamma_{2}} & \frac{l_{2}\sin\left(\beta_{2}-\gamma_{2}-\alpha_{2}\right)}{\sin\gamma_{2}}\\ \frac{\cos(\beta_{3}-\gamma_{3})}{\sin\gamma_{3}} & \frac{\sin(\beta_{3}-\gamma_{3})}{\sin\gamma_{3}} & \frac{l_{3}\sin\left(\beta_{3}-\gamma_{3}-\alpha_{3}\right)}{\sin\gamma_{3}}\\ \frac{\cos(\beta_{4}-\gamma_{4})}{\sin\gamma_{4}} & \frac{\sin(\beta_{4}-\gamma_{4})}{\sin\gamma_{4}} & \frac{l_{4}\sin\left(\beta_{4}-\gamma_{4}-\alpha_{4}\right)}{\sin\gamma_{4}} \end{array}\right]\left[\begin{array}{c} v_{x}\\ v_{y}\\ \omega_{z} \end{array}\right]$$

(2) $$\left[\begin{array}{c} v_{x}\\ v_{y}\\ \omega_{z} \end{array}\right]=T^{+}\left[\begin{array}{c} \omega_{1}\\ \omega_{2}\\ \omega_{3}\\ \omega_{4} \end{array}\right]$$

Table 2 below presents the parameters of the Wasp platform (refer to Figure 5). By substituting and deriving (1) and (2), the inverse kinematics are given in (3) while the forward kinematics are given in (4).

(3) $$\left[\begin{array}{c} \omega_{1}\\ \omega_{2}\\ \omega_{3}\\ \omega_{4} \end{array}\right]=\frac{-1}{r}\left[\begin{array}{ccc} 1 & -1 & -(l_{x}+l_{y})\\ 1 & 1 & \left(l_{x}+l_{y}\right)\\ 1 & 1 & -(l_{x}+l_{y})\\ 1 & -1 & \left(l_{x}+l_{y}\right) \end{array}\right]\left[\begin{array}{c} v_{x}\\ v_{y}\\ \omega_{z} \end{array}\right]$$

(4) $$\left[\begin{array}{c} v_{x}\\ v_{y}\\ \omega_{z} \end{array}\right]=\frac{r}{4}\left[\begin{array}{cccc} 1 & 1 & 1 & 1\\ -1 & 1 & 1 & -1\\ -\frac{1}{\left(l_{x}+l_{y}\right)} & \frac{1}{\left(l_{x}+l_{y}\right)} & -\frac{1}{\left(l_{x}+l_{y}\right)} & \frac{1}{\left(l_{x}+l_{y}\right)}. \end{array}\right]\left[\begin{array}{c} \omega_{1}\\ \omega_{2}\\ \omega_{3}\\ \omega_{4} \end{array}\right]$$

Finally, (5) gives the angular velocity of each wheel, while (6)–(8) provide the longitudinal velocity, transversal velocity and angular velocity of the Wasp platform, respectively.

(5) $$\begin{array}{c} \omega_{1}=\frac{1}{r}\left(v_{x}-v_{y}-\left(l_{x}+l_{y}\right)\omega_{z}\right)\\ \omega_{2}=\frac{1}{r}\left(v_{x}+v_{y}+\left(l_{x}+l_{y}\right)\omega_{z}\right)\\ \omega_{3}=\frac{1}{r}\left(v_{x}+v_{y}-\left(l_{x}+l_{y}\right)\omega_{z}\right)\\ \omega_{4}=\frac{1}{r}\left(v_{x}-v_{y}+\left(l_{x}+l_{y}\right)\omega_{z}\right) \end{array}$$

(6) $$v_{x}\left(t\right)=\frac{r}{4}\left(\omega_{1}+\omega_{2}+\omega_{3}+\omega_{4}\right)$$

(7) $$v_{y}\left(t\right)=\frac{r}{4}\left(-\omega_{1}+\omega_{2}+\omega_{3}-\omega_{4}\right)$$

(8) $$\omega_{z}\left(t\right)=\frac{r}{4(l_{x}+l_{y})}\left(-\omega_{1}+\omega_{2}-\omega_{3}+\omega_{4}\right).$$

In this paper, the $v_{x}$, $v_{y}$ and ω of the Wasp platform are given by the output of FLS, which will be further explained in the latter part. Through inverse kinematics equations, the angular velocity of each wheel is generated according to FLS output, which then drives the platform following the wall according to FLS control.

## 3. Wall-Following Method

### 3.1. Overview

**Figure 6:**
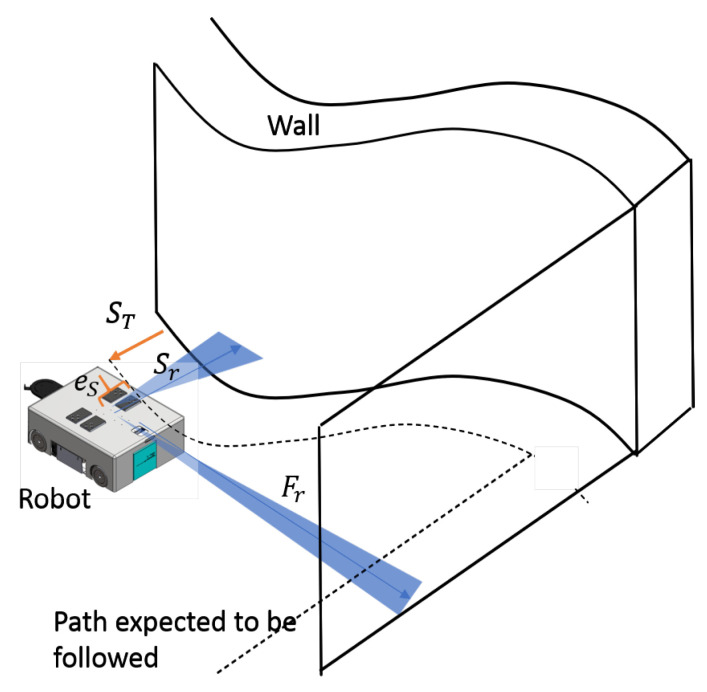
Overview of wall-following.

A scenario of the robot following a curvy wall with a right-handed corner is depicted in Figure 6. It is assumed that the cleaning unit is fixed on the robot’s left side, and the robot follows the wall from the left. The target side clearance for a particular cleaning application and a tool can be defined by the user (denoted as $S_{T}$). The robot is expected to follow a path that has the target clearance distance with the wall throughout the course. The current side clearance range of the robot with the wall is measured from the Lidar sensor as $S_{r}$. In this regard, the minimum reading within a 40° window on the left side is considered. Similarly, the free-range from the front is measured as $F_{r}$, considering a 10° window in the front. The error between the target side clearance (i.e., $S_{T}$) and the current side clearance, measured from the range sensor (i.e., $S_{r}$), $e_{S}$, is calculated as in (9). The robot has to turn toward or away from the wall based on the error to minimize the side clearance error (i.e., if $e_{S}>0$ then ω<0 or vice versa). Furthermore, if there is a front obstruction caused by a wall segment such as a right-turning cornered-wall, the robot has to slow down a bit by altering the front linear velocity ($v_{x}$) and should turn towards the right (i.e., ω<0) to follow the new wall segment. The wall-following of the controller is designed to generate the required controlling actions, $v_{x}$ and ω, to follow any given wall based on the sensory information provided by the Lidar.

(9) $$e_{S}=S_{T}-S_{r}.$$

### 3.2. Fuzzy Logic System (FLS)

A fuzzy logic system can provide quantitative values for a qualitative linguistic input. Mainly, it can map the input for an output space by a set of linguistic rules [62,63]. Fuzzy logic can apply in a situation where the robot perceives the environment data and makes decisions according to the changes in the environment. Further, fuzzy logic is a reliable modelling method for complex robotics systems or systems without realizing specific underlying dynamics [64,65]. The environment and exact dynamics of the robot are uncertain in a situation where the robot is following a wall. Furthermore, these uncertainties increase with the reconfiguration. Expert knowledge of linguistic expressions can be used for the control actions for a wall-following behaviour [66]. Moreover, sensory information obtained from the Lidar readings of the robot may include noises and might not be accurate. According to the literature, fuzzy logic is an excellent solution to provide control actions where the sensory information is vague [67,68] and for unknown environments [66,69]. Therefore, fuzzy logic is used in this paper to achieve the wall-following behaviour for the robot.

**Figure 7:**
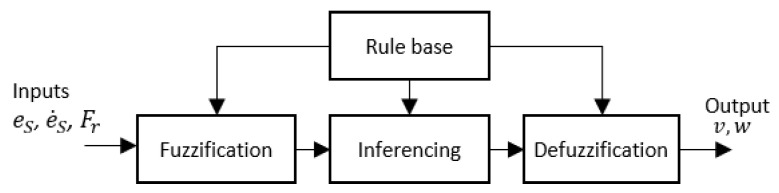
Architecture of Fuzzy logic system.

**Figure 8:**
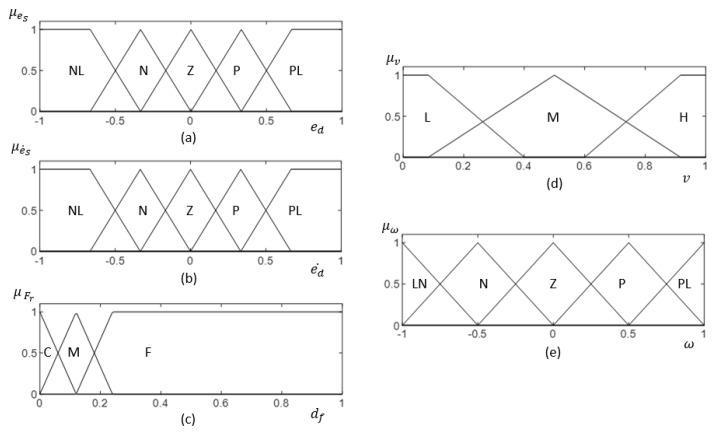
(**a**–**c**) represent the input membership function for $e_{S}$, ${\dot{e}}_{S}$, and $F_{r}$, respectively. (**d**,**e**) show the output membership function for *v* and ω, respectively. Fuzzy labels are defined as NL: ‘Negative Large’, N: ‘Negative’, Z: ‘Zero’, P: ‘Positive’, PL: ‘Positive Large’, C: ‘Close’, M: ‘Moderate’, F: ‘Far’, L: ‘Low’, M: ‘Moderate’, H: ‘High’. The membership functions are defined in normalized scale.

The architecture of the fuzzy logic system for the proposed method is shown in Figure 7. The inputs for the Fuzzy Logic System (FLS) are the side clearance error of the robot (i.e., $e_{S}$), the rate of change of the side clearance error with the wall (i.e., ${\dot{e}}_{S}$), and free front range (i.e., $F_{r}$) for the robot to navigate. Linear velocity (i.e., $v_{x}$) and angular velocity (i.e., ω) are the outputs of the FLS. To cope efficiently with uncertainties, non-singleton fuzzy sets have been specified for the input membership functions in the FLS [70]. Input membership functions are given in Figure 8a–c for $e_{S}$, ${\dot{e}}_{S}$, and $F_{r}$, respectively. The fuzzified values for the inputs are represented as $\mu_{e_{S}}\left(e_{S}\right)$, $\mu_{{\dot{e}}_{S}}\left({\dot{e}}_{S}\right)$, and $\mu_{F_{r}}\left(F_{r}\right)$.

The set of linguistic rules used for mapping the input and output is depicted in Table 3. The output membership functions are given in Figure 8d,e for $v_{x}$ and ω, respectively. Furthermore, the necessary control actions are deduced in this step. The firing strength of *i*th rule, $\alpha_{i}$ can be inferred as given in (10) with max and min operators as t-conorm and t-norm, respectively.

By using the Mamdani implication method, the consequents of the output fuzzy sets corresponding to the *i*th rule can be obtained as in (11). Here, $\mu_{v_{x_{i}}^{\prime }}$ and $\mu_{\omega_{i}^{\prime }}$ are the fuzzy consequents of the outputs $v_{x}$ and ω, respectively. The fuzzy aggregation operator, max, is used to combine these fuzzy consequents into a single set for each output as given in (12).

The FLS should provide crisp control outputs to control the robots reference linear and angular velocities ($v_{x}$ and ω) for the effective wall-following cleaning. These crisp outputs could be obtained by (13) using the center of area method for the defuzzification.

(10) $$\alpha_{i}=\min\left\{\mu_{e_{S_{i}}}\left(e_{S}\right),\mu_{{\dot{e}}_{S_{i}}}\left({\dot{e}}_{S}\right),\mu_{F_{r_{i}}}\left(F_{r}\right)\right\}$$

(11) $$\begin{array}{c} \mu_{v_{x_{i}}^{\prime }}\left(v_{x}\right)=\min\left\{\alpha_{i},\mu_{v_{x_{i}}}\left(v_{x}\right)\right\}\\ \mu_{\omega_{i}^{\prime }}\left(\omega \right)=\min\left\{\alpha_{i},\mu_{\omega_{i}}\left(\omega \right)\right\} \end{array}$$

(12) $$\begin{array}{c} \mu_{v_{x}^{\prime }}\left(v_{x}\right)=\max\left\{\mu_{v_{x_{1}}^{\prime }}\left(v_{x}\right),\mu_{v_{x_{2}}^{\prime }}\left(v_{x}\right),\ldots ,\mu_{v_{x_{i}}^{\prime }}\left(v_{x}\right),\ldots ,\mu_{v_{x_{N}}^{\prime }}\left(v_{x}\right)\right\}\\ \mu_{\omega^{\prime }}\left(\omega \right)=\max\left\{\mu_{\omega_{1}^{\prime }}\left(\omega \right),\mu_{\omega_{2}^{\prime }}\left(\omega \right),\ldots ,\mu_{\omega_{i}^{\prime }}\left(\omega \right),\ldots ,\mu_{\omega_{N}^{\prime }}\left(\omega \right)\right\} \end{array}$$

(13) $$\begin{array}{c} v_{x}*=\frac{\int v\mu_{v_{x}^{\prime }}\left(v_{x}\right)dv}{\int \mu_{v_{x}^{\prime }}\left(v_{x}\right)dv_{x}}\\ \omega *=\frac{\int \omega \mu_{\omega^{\prime }}\left(\omega \right)d\omega }{\int \mu_{\omega^{\prime }}\left(\omega \right)d\omega }. \end{array}$$

## 4. Results and Discussion

### 4.1. Experiment Setup

**Figure 9:**
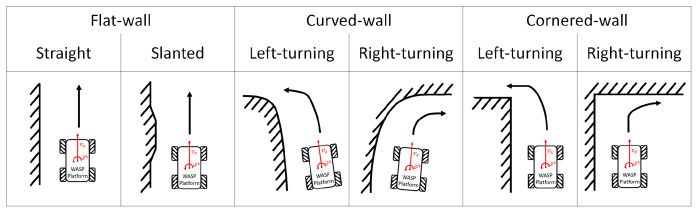
Different types of wall characterization considered for the experiment.

In order to examine the wall-following behaviour, experiments were conducted with different types of wall conditions using the same Wasp platform. Throughout these experiments, the Wasp platform was following the wall on its left, and $S_{T}$ was maintained at 80 cm unless further indicated for the ease of data analysis and the comparison between different experiments. Three types of walls—as shown in Figure 9, the flat-wall, the curved-wall and the cornered-wall—were used to demonstrate the wall-following behaviour of the Wasp platform. Although flat walls and right corners are most common, variations of walls are important to be considered for it to be pragmatically implemented in an actual hospital. Unconventional architectural design is not absent in the modern world; hence it improves the autonomy of robots. Doors can be treated similarly to flat walls, but transparent features are not detectable by LiDAR. In this proposal, the exploration of a suitable sensing method is not part of the scope of this study.

To qualify the algorithm’s function, the input data will evidently show that the corresponding output data must react based on the intended logic of the algorithm. Secondly, the physical platform must not encounter any collision in its working environment. Lastly, the values of the logged data are evaluated against a threshold for its fault tolerance. The error tolerance is determined by the reach of the cleaning module. These aspects of the robot and the algorithm will serve as qualifiers to the system.

The conditions of the experiment will be set by the characterisation of the wall, the initial side clearance from the wall plane and set desired side clearance. First, during flat-wall experiments, the impact of the initial robot position was investigated by locating the platform at a distance away from a straight flat-wall, the same as $S_{T}$, further than $S_{T}$ and closer than $S_{T}$, respectively, at the beginning of the experiments. More experiments were then conducted at a slanted flat-wall to observe the wall-following behaviour when there is a change of angle at the wall. Next, for both curved-wall and cornered-wall, experiments on both left-turning and right-turning directions were conducted. Lastly, experiments were carried out with $S_{T}$ at 100 cm and 60 cm to verify the robot wall-following ability at different $S_{T}$. The validation of these experiments assumes that the parameters of the FLS are suitably tuned and adjusted based on the given platform and operating environment. The experimental data and analysis are presented in the following section. An explanatory video that demonstrates the experiments is given as a multimedia attachment in Supplementary Materials.

### 4.2. Results Analysis

**Figure 10:**
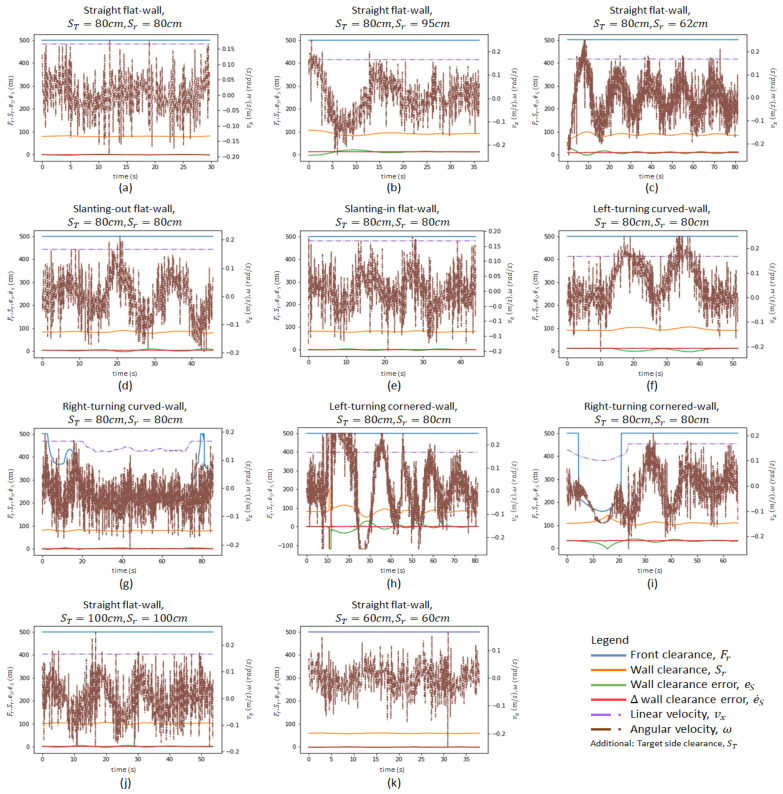
Variation of crucial parameters of the controller. (**a**–**c**) show flat-walls experiments with different initial positions, (**d**,**e**) for slanting-wall experiments, (**f**,**g**) for curved-wall experiments, (**h**,**i**) for cornered-wall experiments, (**j**,**k**) for flat-wall experiments with different target side clearance.

**Figure 11:**
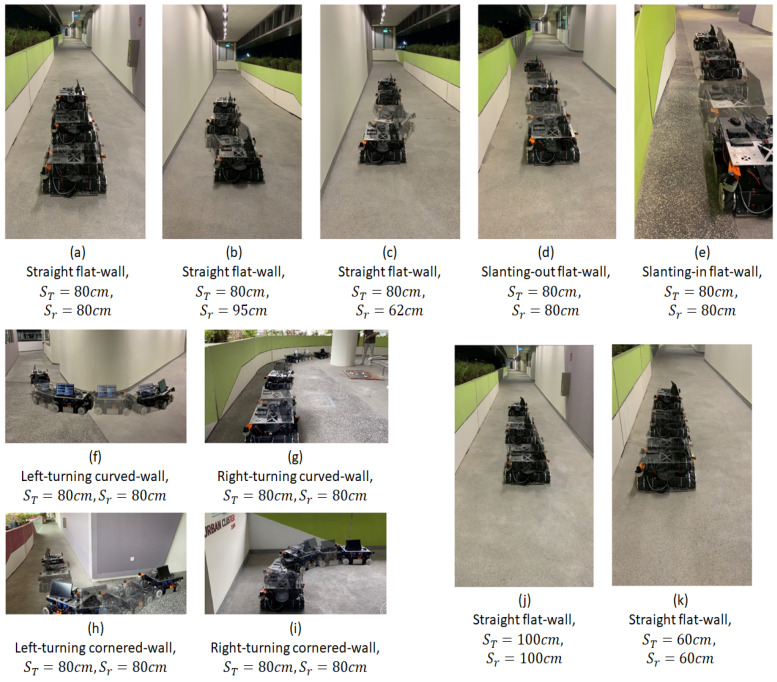
Robot motion trajectory in seven-frame time-lapse illustration. (**a**–**c**) show flat-wall experiments with different initial positions, (**d**,**e**) for slanting-wall experiments, (**f**,**g**) for curved-wall experiments, (**h**,**i**) for cornered-wall experiments, (**j**,**k**) for flat-wall experiments with different target side clearance.

The first set of experiments was conducted to observe the effect of the robot’s initial position on its wall-following behaviour. When the initial robot position was significantly close to the intended $S_{T}$, the platform was able to perform the wall-following with $e_{S}$ oscillating within the range of ±3 cm as shown in Figure 10a. The oscillations were considerably small (maximum = 2.5 cm, average = 0.95 cm). These oscillations are considered insignificant for the wall cleaning applications as these oscillations can be coped with by the end-effector. Figure 11 shows the robot’s motion trajectory in seven-frame time-lapse illustrations for all the experiments. From Figure 11a, we can see that the robot’s trajectory first encountered minor swaying and was then able to proceed in a stable trajectory along the wall. In contrast, two cases were considered with the initial robot position offset away from $S_{T}$, with one case set further away from the wall at 95 cm and the other set closer to the wall at 62 cm. Both measurements are relative distances from the wall to the robot base frame. As shown in Figure 11b,c, the platform was oscillating about $S_{T}$ from the beginning of both experiments. These oscillations gradually decreased in magnitude and converged towards zero. As shown in Figure 10b,c, the robot’s angular velocity, ω, fluctuated when $e_{S}$ was large and slowly stabilised towards zero as $e_{S}$ converged towards zero. This data trend proves that the wall-following behaviour is well regulated by $e_{S}$ to achieve $S_{T}$ through manipulating the robot’s angular velocity, ω. The platform oscillated between 6.5 cm and 3.5 cm for slant-in and slant-out flat-walls in the experiments conducted with slanted walls, respectively. On average, the oscillations were 2.6 cm for the slant-in flat-wall and 1.5 cm for the slant-out flat-wall. The experimental data for slanting-walls are presented in Figure 10d,e. Figure 11d,e show that both experiments encountered relatively large oscillations when the robot was transiting between different wall surfaces. However, these oscillations progressively stabilised to a stable trajectory along the wall. These experiments evidently verified that the platform’s wall-following behaviour is able to operate on straight flat-walls and flat-walls with slanted features.

For curved-wall experiments, robot wall-following behaviours at left-turning and right-turning curved-walls were both considered. As shown in Figure 10f, the robot’s angular velocity, ω, increased to 0.2 rad/s when $e_{S}$ was at negative values. Given that $e_{S}=S_{T}-S_{r}$, a negative value $e_{S}$ indicated that the Wasp platform was further away from the wall than $S_{T}$, thus inducing a positive angular velocity, ω, to drive the robot towards the left. Figure 11f shows the Wasp platform was turning left, following the wall. After navigating past the curved feature, the platform was able to follow the wall at $S_{T}$ (preset at 80 cm). On the other hand, the experimental data for a right-turning curved-wall are shown in Figure 10g. As the robot turned right, the targeted wall segment was perceived as an obstacle in front of the robot. It is evident in the graph (Figure 10g) that the front clearance decreased, which led to a reduction of the robot’s velocity, $v_{x}$. Concurrently, a negative angular velocity, ω, was induced to turn the robot clockwise to avoid crashing into the wall in front. The gradual variation of $e_{S}$ caused a relatively stable angular velocity, ω. Figure 11g shows the Wasp platform proceeded in a right turning trajectory following the right-turning curved-wall. The outcome of these two experiments validates the wall-following capability in managing curved-walls.

The cornered-wall experiments time-lapse illustrations are shown in Figure 11h,i. The experimental data in Figure 10h show that the behaviour of the robot’s linear velocity, $v_{x}$, during the left-turning cornered-wall experiment is similar to that in the left-turning curved-wall experiment. The robot’s linear velocity, $v_{x}$, was constant as there was no obstruction detected in front of the robot while the robot’s angular velocity, ω, induced by the variation of $e_{S}$, played a role in regulating $S_{r}$ according to $S_{T}$. For the right-turning cornered-wall, the experimental data shown in Figure 10i show that as $F_{r}$ decreased when the robot detected the wall segment in front as an obstruction, the robot linear velocity, $v_{x}$, decreased to slow down the robot’s movement. At the same time, the angular velocity, ω, was at a negative value to drive the robot towards the right even though $e_{S}$ was at a negative value. This phenomenon is caused by the rules defined in fuzzy logic where priorities are appropriately set to react to $F_{r}$ before $S_{r}$ for collision avoidance.

In addition, to ensure the robot wall-following can handle different $S_{T}$, experiments were conducted with $S_{T}$ at 100 cm and 60 cm, respectively, with flat-walls. Figure 10j,k show the experimental data, which are similar to those of the experiment with $S_{T}$ set at 80 cm. Figure 11j,k show that the platform followed the wall with $S_{T}$ set at 100 cm and 60 cm, respectively. These experiments proved that the wall-following algorithm is operational at different $S_{T}$, which increases its flexibility for various mounted equipment.

In general, the proposed controller was capable of following various common walls, as seen in the experiments above. The different experimental conditions have covered most of the possible scenarios in real life. As the straight flat-wall experimental results show, the controller performed the best with the least oscillation when initial $S_{r}=S_{T}$, therefore it is advisable to start the cleaning operation of a wall cleaning robot after it moves to the preset wall clearance. The other experiments, which included slanting flat-wall, curved-wall and cornered wall, on the other hand, showed the versatile adaptability of the robot on different types of wall features. Hence, the proposed FLS controller is suitable to utilize for wall-following robots as the experimental results above qualify its capabilities.

### 4.3. Discussion

The existing wall following methods based on PID controllers [41,42] are only effective on straight walls. In contrast, experimental results obtained by deploying the robot to slanted, curved, and cornered walls (i.e., cases (d)–(i)) showed the effectiveness of the proposed controller for non-straight walls. Furthermore, FLSs [45,46,47,48,49,50,51,52,53,54] used in the existing methods are designed to work with a specific reference distance, where the FLSs have to be tuned or tailored to work with various reference distances. On the other hand, the method proposed in this paper is capable of working with various reference distances without requiring modifications. This ability of the controller could be noted from the experiment cases (a), (j), and (k), where the robot was operated with three different reference distances. These facts revealed the superiority of the proposed controller over the existing methods.

Quantitative comparison of the experimental results of the method against the results of the existing method could provide many insights. To perform a proper quantitative comparison, the existing methods considered for the comparison should be implemented, and the same set of experiments should be conducted considering all the methods. However, in this particular case, such a comparison was not feasible since the FLSs based on expert knowledge (e.g., [51,52,53,54]) have been designed to work on a specific reference distance with a wall and a common reference distance across all the methods could not be considered for performing a proper comparison. Furthermore, some of the existing methods utilize parameters specific to the hardware platform. For example, the outputs of the controllers proposed in References [51,52] are defined as the PWM for the motors. In some cases, the methods require robot platforms with specific sensor arrangements that could not be facilitated in the hardware platform used in this work. Thus, such methods could not be mapped to the robot platform used in this work for reimplementation. These were the reasons for limiting the comparison to a qualitative comparison.

**Figure 12:**
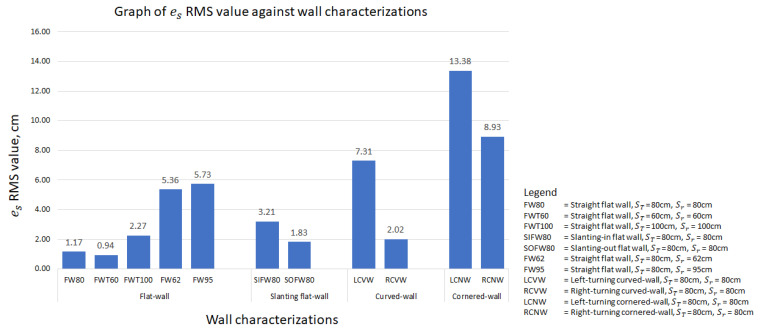
Graph of $e_{s}$ RMS value against different wall characterizations.

In this study, the paper considers all types of wall that a robot frequently encounters and/or might encounter. A graph of $e_{s}$ root mean square (RMS) value against different wall characterizations is plotted in Figure 12 to provide a clearer insight into the performance of the FLS proposed in this paper. The wall characterizations considered in the experiments are categorized into flat-wall, slanting flat-wall, curved-wall and cornered-wall. In the flat-wall category, the RMS value is generally below 2.5 cm. Exceptions are found in cases FW62 and FW95, yet this is explainable as these two experiments were begun with an error to prove the capability of the proposed FLS in wall-following behaviour. As shown in Figure 10b,c, $e_{s}$ converged to zero towards the end of the experiments. In the slanting flat-wall category, the RMS value is relatively high compared to FW80, FWT60 and FWT100 in the flat-wall category. This is due to the variation of $e_{s}$ when the robot platform encountered a change of wall angle (see Figure 10d,e) and eventually $e_{s}$ converges to zero towards the end of experiments. Thus, it can be concluded that, for flat-wall characterization including slanting flat-wall, the proposed FLS is capable of, firstly, correcting the distance between the wall and the robot platform from an error distance to the target distance, and secondly, controlling the robot platform to follow the wall at the desired target distance with an error distance of not more than 5 cm.

For curved-walls and cornered-walls, the RMS value shows an increasing trend compared to flat-walls. Throughout both curved-wall and cornered-wall categories, left-turning cases have a higher RMS value compared to the right-turning cases. This is due to the working concept of the FLS and the position of the end-effector. In this paper, the end-effector is assumed to be attached to the left side of the robot platform. Hence, the proposed FLS will always attempt to drive the robot following the left side wall with a preset target distance. In such a configuration, $e_{s}$ always tends to increase during a left-turning case and decrease during a right-turning case. The robot platform also tends to travel with a slower velocity $v_{x}$ in right-turning cases due to the detection of the wall segment in front as an obstruction, which allows more time for the robot platform to react to $e_{s}$. On the other hand, cornered-wall cases have the highest RMS value among all cases as the robot platform does not travel entirely according to the wall characterization. Instead of following the wall in a 90° trajectory turn at the corner, the robot platform travelled in an arc trajectory as shown in Figure 11h,i. Eventually, a large $e_{s}$ value was generated at the corner position and contributed to the high RMS value. Future work is to be carried out to further adapt the proposed FLS to cornered-walls.

Overall, it has been shown that the proposed FLS is capable of driving the robot platform following the wall with an $e_{s}$ RMS value of not more than 10 cm. This $e_{s}$ RMS value can be coped with by means of end-effector, for example, a preloaded spring system, to ensure the end-effector is in contact with the wall during the disinfection process. At the same time, it has also been shown that the proposed FLS is capable of regulating the $e_{s}$ value towards zero when the robot platform encounters a change in the wall arrangement, for example, a curved-wall or a cornered-wall.

## 5. Conclusions

In the current SARS-CoV-2 pandemic crisis, autonomously operated robots are in high demand. Robots are known to effectively improve the containment of viruses and reduce the risk to frontline and healthcare workers by disinfecting high-risk environments. The targeted area in high-risk environments, such as airports, public transport areas and hospitals, was identified as being the walls of indoor environments. This paper introduces a novel application in inter-reconfiguration and a suitable design of autonomous control for wall disinfection. The inter-reconfigured cleaning modules may vary. Wall-following abilities are considered advantageous for the adoption of autonomous machines. This addresses the demands of disinfecting the public environment. The mounted end-effector can vary from anti-viral sprayers, UV-C lamps to mechanical cleaning mechanisms. For a robot to perform efficaciously and to adequately engage with such equipment properly, the navigating motion trajectory of the robot base must be able to move along the surface of a wall at a prescribed distance that is to be maintained. This paper presented the development of a reconfigurable wall cleaning mobile robot equipped with wall-following capability. Wasp has demonstrated the application of inter-reconfiguration for disinfection purposes and validated the physical development of FLS wall-following capabilities.

Wasp is a highly versatile robot platform that is currently under development for healthcare settings. Wasp will be utilized with various end-effectors providing flexibility in the choice of disinfection method. The wall following algorithm is portable, such that a typical mobile robot can adapt the algorithm for wall-following. In this study, the integration of real-world applications was enabled by the functions of inter-reconfiguration and the FLS wall-following algorithm cohesively. This system can help manage public health and safety during the current pandemic and for future safeguarding. The robot is not limited to use in hospitals but could also be used in other public environments regardless of risk levels. The platform has adopted the proposed FLS wall-following behaviour by reading front and side clearances alongside numerically processed errors. With proper adjustments of the algorithm’s sensitivity, the platform is proven to be capable of wall-following with seven types of different wall features and has additionally been tested with varying offsets for its initial starting point. The robot was successful in managing its trajectory even with different targeted side clearances.

Throughout the experiments, the oscillating errors of the robot trajectory encountered are reasonable and considered negligible. The platform has fulfilled the promise of versatility. Real-world implementation of the proposed wall-following method was verified and proven in the scope of this research. The evaluation of the cleaning efficiency and energy usage of the robot is to be explored in future work.

## Figures and Tables

**Table 1:** Limitations of the existing wall following methods.

Method	Major Limitations
Line tracing methods proposed in [34,35]	Clear visible lines have to be drawn on the floor, which is not convenient.
PID controllers proposed in [41,42]	Designed only for straight walls and the methods are ineffective in slanted or curved walls that a disinfection robot can often encounter.
Neural network based methods proposed in [43,44]	Validation is limited to offline classification performance tests with a dataset; neither experiment nor simulations were conducted for the validation.
FLS tuned through metaheuristics [45,46,47,48,49,50]	Retraining is required in the case of changing the reference distance with a wall. For a robot that uses various disinfection tools, it is essential to have the flexibility to change the reference distance.
FLS proposed in [51,52,53,54]	The methods are designed to maintain only a specific reference distance with a wall. In the case of altering the reference distance, the membership functions of the FLS should be redesigned. Thus, the methods are not convenient for a robot intended to operate with various reference distances.
FLS proposed in [55]	Validation is limited to simulations using a generic robot model.

**Table 2:** Parameters of the Wasp platform.

*i*	$\alpha_{i}$	βi	γi	$l_{i}$	$l_{\mathrm{ix}}$	$l_{\mathrm{iy}}$
1	$\frac{\pi }{4}$	$\frac{\pi }{2}$	$-\frac{\pi }{4}$	*l*	$l_{x}$	$l_{y}$
2	$-\frac{\pi }{4}$	$-\frac{\pi }{2}$	$\frac{\pi }{4}$	*l*	$l_{x}$	$l_{y}$
3	$\frac{3\pi }{4}$	$\frac{\pi }{2}$	$\frac{\pi }{4}$	*l*	$l_{x}$	$l_{y}$
4	$-\frac{3\pi }{4}$	$-\frac{\pi }{2}$	$-\frac{\pi }{4}$	*l*	$l_{x}$	$l_{y}$

**Table 3:** Rule base of the FLS.

$F_{r}$ = F	$\dot{e_{S}}$\$e_{S}$	NL	N	Z	P	PL
	NL	ω = PL *v* = H	ω = PL *v* = H	ω = PL *v* = H	ω = P *v* = H	ω = Z *v* = H
	N	ω = PL *v* = H	ω = PL *v* = H	ω = P *v* = H	ω = Z *v* = H	ω = N *v* = H
	Z	ω = PL *v* = H	ω = P *v* = H	ω = Z *v* = H	ω = N *v* = H	ω = NL *v* = H
	P	ω = P *v* = H	ω = Z *v* = H	ω = N *v* = H	ω = NL *v* = H	ω = NL *v* = H
	PL	ω = Z *v* = H	ω = N *v* = H	ω = NL *v* = H	ω = NL *v* = H	ω = NL *v* = H
$F_{r}$ = M	ω = N *v* = M					
$F_{r}$ = C	ω = NL *v* = L					

## Supplementary Materials

The following are available online at https://www.mdpi.com/article/10.3390/s21186096/s1.
